# The Alcohol Question in England

**Published:** 1879-02

**Authors:** 


### ARTICLE V.

### *The Alcohol Question in England.*

Within a comparatively recent time serious attempts at temperance reform have been begun in England. Many prominent persons have interested themselves in the agitation, which has shown itself in the organization of coffee-house companies, in the securing of pledges, and in demands for new legislation. The idea of drinking only water is a novel one to the English mind, but the energetic efforts of the reformers have at length brought it into prominence. There has recently appeared in the *Contemporary Review* a kind of "symposium" on the alcoholic question, the contributors being seven English physicians, most of whose names are familiar. Some idea of what their opinions are may not be uninteresting.

Sir James Paget takes the ground that the moderate habitual use of alcohol is "certainly pleasant and probably useful." In regard to moderate drinking, he says that the balance of medical and of popular feeling favors it, and that neither statistics nor physiological or pathological researches have proved it injurious. Nations who use alcohol largely, compare well with those who do not use it, and they do not appear to have inherited evils from their many generations of drinking ancestors.

Dr. T. Lauder Brunton takes up the more practical side of the question, discussing how and when alcohol is useful. There is, he says, a small class to whom alcohol is a poison ; the smallest amount sets them wild. There is a second class whom alcohol exhilarates and quickens for the time ; such persons indulge in it at great risk. The great majority of persons under middle age do not need it, and as a rule, are better without it. In persons who are in the decline of life, however, and in the debilitated, alcohol is a powerful and beneficial remedy. Alcohol is given as a food and as a stimulant. It is a food, but is one which interferes with the oxydation of other foods in the body while it is being itself decomposed, and as a food it is only adapted to febrile conditions. As a stimulant it acts directly upon the heart, and reflexly upon the stomach, stimulating the circulation of the brain. After the first stimulus to the nervous system, the succeeding effect of alcohol is one of progressive paralysis. The higher centres suffer first, notably the judgment, and finally all succumb. Alcohol as a stimulant is useful occasionally to tide over a severe crisis, but its best effect is in rousing the system at the close of exhausting work.

Dr. Albert J. Bernays believes in the moderate use of alcohol also. He dwells more especially on the causes and extenuating circumstances of intemperance. In regard to these, he says that the water furnished by London, to its lower classes at least, is extremely bad and undrinkable. Then the adulterations in beer make its effect worse. Sugar is put in, and this destroys its thirst-quenching property, and salt acts in the same way; these being the two important adulterations. The variations in the alcoholic strength of liquors increase intemperance. At present, gin may have all the way from fifty-four to eighty per cent. of alcohol in it. The atmosphere of public-houses is foul and overheated, and is injurious to the workingmen who sit there. Beer is the best form of alcoholic drink according to Dr. Bernays, and wine the next. The present intemperance cannot be corrected by teetotalism, but it can be alleviated by other methods.

Dr. Walter Moxon takes ground against total abstinence, but devotes most of his article to a psychological explanation of why a man becomes a sot. His analysis of the question is sufficiently profound and correct, but it only tells us in pollysyllabic terms that the nervous, excitable temperaments are more susceptible to alcohol than the phlegmatic ones.

Dr. S. Wilkes asserts alcohol to be, to all intents and purposes, a narcotic and not a stimulant. It does not help those who are under special mental pressure, such as students working for prizes. It makes those engaged in intellectual effort less clear-headed, and under its influence the English laborer does less work.

Sir Wm. Gull is more careful in his recommendations of alcohol. In disease and debility it is useful, and also in overwork; but in the latter instance other things will do just as well, and Gull himself, when exhausted, eats raisins instead of drinking wine. Good food will supply all the wants of the system up to middle life, and though a glass of beer may help a laborer along, a biscuit will do just as well. Intellectual work can be done better without the alcohol. Bitter tonics or Liebig's extract of meat may quiet the craving for liquor which many persons have at times.

Dr. C. Murchison states that a man in good health does not need alcohol, and is probably better without it. He may take liquor occasionally without harm, but its habitual use, even in moderation, is attended with risk and may even induce disease. In conditions of the system characterized by weakness of the circulation, the habitual daily use of alcohol is likely to be beneficial.

It will be seen that in general these views coincide with those of the profession at large. Alcohol in disease is a valuable remedy; in the decline of life it is a useful adjunct to the diet; in healthy persons who have been overworked, it helps recuperation; its habitual use is always attended with risk.

As regards total abstinence, we believe it to be unattainable, and, except for the young and healthy, undesirable. There is an appetite for alcohol which will be satisfied, and which neither temperance societies nor legislatures can destroy. It would be better, then, if the spasmodic efforts of these bodies to prevent the use of alcohol altogether were directed to seeing that it is used temperately. There is much to be done in the way of introducing good malt liquors and light wines, in establishing coffee-houses, and in introducing harmless substitutes for alcohol. It might be of help if physicians would impress the fact that alcohol is essentially a narcotic, not a stimulant. And something might be accomplished by educating every one, not omitting the higher classes, to a deeper sense of the beastliness of inebriety.—*Medical Record.*

